# Spinal cord stimulation for refractory pericarditis: a case report and a review of the mechanism of action

**DOI:** 10.3389/fpain.2023.1174044

**Published:** 2023-07-05

**Authors:** Simon Elsliger, Jacob Saucier, Andre Schneider, Antonios El Helou

**Affiliations:** ^1^ Centre de Formation Medicale du Nouveau Brunswick, Universite de Sherbrook, Moncton, NB, Canada; ^2^ Department of Anesthesia and Pain Medicine, Vitalite Health Network, Bathurst, NB, Canada; ^3^ Division of Neurosurgery, Horizon Health Network, Moncton, NB, Canada; ^4^ Faculty of Medicine, Memorial University of Newfoundland, St. John’s, NL, Canada; ^5^ Faculty of Medicine, Dalhousie University, Halifax, NS, Canada

**Keywords:** spinal cord stimulation, pericarditis, heart failure, refractory chest pain, mechanism of action

## Abstract

**Background and objectives:**

In recent years, spinal cord stimulation (SCS) has emerged as a promising management option for chronic pain of multiple etiologies. While its effectiveness has been strongly suggested in many patients, the exact mechanism of action of SCS is incompletely understood. This article reviews the leading mechanisms underlying the analgesic and cardiovascular effects of SCS and reports its novel benefits in a case of recurrent pericarditis.

**Literature review:**

Throughout history, the analgesic properties of SCS were thought to arise via stimulation of the spinothalamic tract. Although this mechanism has been thoroughly reported, new research and patient outcomes from SCS have revealed various additional properties that cannot be fully explained by this mechanism alone. Evidence suggests that SCS enhances calcitonin gene-related peptide release and modulates inflammatory cytokine secretion, sympathetic tone, and inhibitory neurotransmitter secretion. These distinct mechanisms likely collectively contribute to the therapeutic effects of SCS on the cardiovascular system and pain management.

**Case report:**

We report the case of a 48-year-old male patient with recurrent pericarditis, characterized by refractory angina-like pain and reduced left ventricular ejection fraction (LVEF). After 1 year of having a spinal cord stimulator implanted, the patient is free from pain and narcotics, with a reduction of 428 mg equivalent dose of morphine. The patient's LVEF increased from 40% to 45% without changes to his previous medical treatment. This is the first reported case of refractory pericarditis managed with spinal cord stimulation.

**Conclusion:**

Recognizing the improved pain management, reduced narcotic usage, and improved LVEF in our patient following SCS is critical to paving the way toward a complete understanding of the mechanism of action of SCS. This case reveals the therapeutic potential of SCS for cardiovascular pathologies other than refractory angina pectoris.

## 1. Introduction

In 1967, spinal cord stimulation (SCS) was introduced to the field of medicine by Shealy et al. for the treatment of chronic intractable pain in an oncological patient at Case Western Reserve University (1). Since its arrival in the therapeutic arsenal of neurosurgeons, SCS has been seen as a safe and effective method to manage pain of multiple etiologies such as complex regional pain syndrome (CRPS) types I and II, failed back surgery syndrome, unmanageable leg and low back pain, neuropathic pain, and refractory angina (RFA) pectoris (2–6). Although SCS has been in use for the majority of the past five decades, its application for the management of refractory anginal pain only surfaced in 1987, as reported by Murphy et al. (7). SCS for refractory angina has been proven to reduce chronic anginal pain and improve quality of life (6, 8). The clinical presentation of refractory chest pain is not exclusive to coronary heart disease, as angina-like pain can originate from numerous pathologies (9). Non-vascular heart pathologies such as pericarditis may cause chronic, refractory chest pain (10). In fact, 30% of patients who experience one episode of acute pericarditis go on to experience recurrent events of pericarditis chest pain (10). The standard treatment for patients living with recurrent pericarditis involves aspirin and/or non-steroidal anti-inflammatory drugs and colchicine. However, in cases where some patients do not respond to this treatment, a short course of prednisone or anti-IL-1 biological agents may be beneficial in controlling the condition (11). Heart failure (HF) associated with pericarditis may be present in patients with myocardial involvement. Mild to moderate left ventricular systolic dysfunction [left ventricular ejection fraction (LVEF) of 40%–50%] occurs in 13%–15.4% of patients with perimyocarditis, with recurrence rates of 12%. Left ventricular dysfunction typically resolves within 12–36 months following an acute event (11). Although refractory chest pain has multiple etiologies, SCS has only been reported in the treatment of refractory angina pectoris (10). Our literature review reveals evidence supporting the efficiency of SCS in treating cardiovascular conditions, including refractory angina pectoris and heart failure (12, 13). In this report, we present a novel case of refractory chest pain and heart failure related to recurrent pericarditis that did not respond to maximal medical therapy, along with a review of the mechanism of action of SCS in cardiovascular pathologies.

## 2. Case report

We report a case of a 48-year-old Caucasian male patient with a past medical history of type II diabetes mellitus, obesity, dyslipidemia, chronic obstructive pulmonary disease, and hypertension. He was initially referred to our multidisciplinary clinic for chronic lower back pain. The clinical evaluation revealed a 3-year history of chest pain caused by multiple recurrent pericarditis of unknown origin.

The patient underwent multiple para-clinical evaluations for his pericarditis, with no determined etiology. The initial transthoracic ultrasound showed a reduced ejection fraction at 33% with moderate hypokinesis of the left ventricle and left ventricular asynchrony. Subsequently, ventriculography was performed, which showed severe hypokinesis of the left ventricle, with an associated ejection fraction of 26% and no signs of mitral insufficiency. A normal coronary angiogram excluded coronary heart disease as the culprit of heart failure. Rheumatological evaluations and a search for auto-immune diseases were performed, which did not reveal any underlying disorders. With regard to *lombalgia* investigations, neuraxial and sacroiliac joint MRI showed evidence of facet arthritis at the levels of L3–L4, L4–L5, and L5–S1, but no signs of canal stenosis, bone lesions, or indications of ankylosing spondylitis were observed.

Our multidisciplinary pain clinic evaluated the patient for his persistent low back pain. Initially, there was no indication for spinal surgical intervention. Instead, medical treatment with interventional spine procedures was offered. Over a period of 4 years and 7 months, hydromorphone was titrated up to a maximal equivalent dose of morphine of 428 mg. Gabapentin was initiated, which was then discontinued due to the absence of a therapeutic response. Pregabalin was also tried, but the patient reported dizziness and drowsiness. Regarding co-analgesia, he was prescribed maximal doses of acetaminophen and duloxetine at 90 mg/day. With regard to his chest pain, the medical treatment for pericarditis was optimized, which improved his left ventricle ejection fraction from 26% to 40%. However, no significant reduction of his severe chest pain ensued, rated as 10/10 on the visual analog scale (VAS) despite maximal medical therapy including scheduled narcotic intake. At this point, a stellate ganglion block (SGB) was performed by an interventional pain physician, granting temporary pain relief as measured by a VAS score of 5/10, without changes in the medical management.

After a comprehensive evaluation of the patient, he was determined as an ideal candidate for SCS to address his chronic lower back pain. Due to his history of refractory pericarditis and chronic chest pain, the pain physician and neurosurgeon jointly decided to offer him the option of placing the stimulator at the cervicothoracic junction instead of the lower thoracic level. The decision aimed to target and potentially alleviate his chest pain and lower back pain. The risks of the operation and the likelihood of trial failure were thoroughly discussed. In the event of failure of the initial procedure, the possibility of a second surgical intervention to reposition the epidural paddle to the low thoracic spine was also addressed. These discussions took place separately at the pain clinic and the neurosurgeon's office. The patient was subsequently evaluated during a multidisciplinary round, and after considering all information, he provided consent for the procedure.

**Figure 1:**
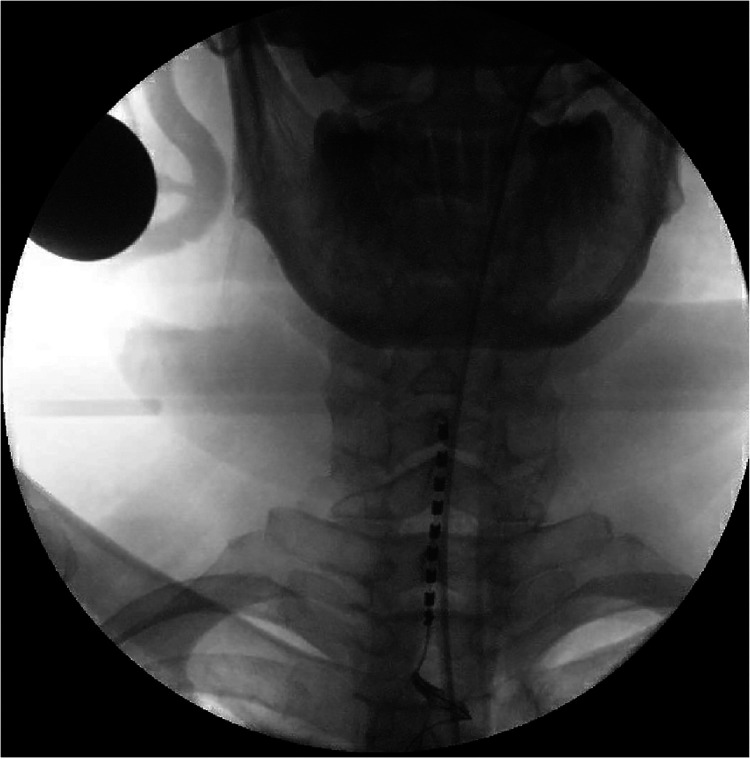
Preoperative anteroposterior view of the epidural Lamitrode® eight-contact paddle inserted through a laminotomy at the T2–T3 level and advanced under fluoroscopy guidance to have the first contact at the level of the C5–C6 intervertebral disc.

A 14-day trial period of SCS was conducted using an eight-contact Lamitrode® paddle with bipolar stimulation, provided by Abbott and inserted through an interlaminar approach (Figure 1). The configuration was optimized after multiple trials of changing the contact levels performed mainly on days 0 and 1. The programming utilized the burst mode, with 500 ms of pulse width, 50 Hz of frequency, and 0.8–1 mA of stimulation with cycling (Abbott®). During the trial, the patient reported a 60% reduction in chest pain and an 80% reduction in back pain. There was also an improvement in his sleeping pattern, with uninterrupted 5 h of sleep per night compared with his previous pattern of 1–2 h, and a reduction of equivalent dose of morphine by 164 mg, from 428 to 264 mg of daily morphine equivalent dose. Sham stimulation was conducted between days 5 and 7 of the trial period. Following the patient's evaluation, a reprogramming session was conducted, maintaining the same stimulation frequency and pulse width but setting the intensity to 0. On day 8, a clinical reassessment was performed to determine success, which is determined by the recurrence of pain or the indication of ineffectiveness compared with the initial 5-day period. This strategy has been used since the inception of our neuromodulation program and has been documented in the literature (14, 15).

Following a successful trial period and sham stimulation test, the spinal cord stimulator was internalized using a Proclaim internal pulse generator provided by Abbott® while maintaining the burst programming parameters. The initial follow-up was virtually conducted after 2 months, where the patient tested positive for COVID-19 and was hospitalized.

At 6 months post–SCS implantation, a transthoracic cardiac ultrasound performed on the patient showed an increase in ejection fraction to 45% despite having no changes in his previous medical treatment, and he became narcotic-free.

During the 8- and 12-month follow-ups, the patient reported consistent pain control without needing any narcotic intake. In addition, the pain relief allowed him to initiate an exercise program for weight loss.

## 3. Literature review

### 3.1. Physiological mechanism of SCS in refractory angina

RFA is a crippling condition marked by angina that is unresponsive to standard therapies for coronary artery disease, such as medications including nitrates, calcium-channel blockade, β-adrenoceptor blockade, and vasculoprotective agents, as well as procedures such as percutaneous coronary intervention and coronary artery bypass grafting (16). People with this condition experience daily pain and typically have a reduced quality of life, emphasizing the value of SCS introduction to alleviate pain in patients living with RFA despite maximal medical therapy (17). Indications for SCS include (1) patients living with severe refractory angina pectoris who are ineligible for coronary revascularization surgery due to risks of surgical complications and (2) patients who do not respond to standard treatments for RFA, which can vary across different centers (17–19). Contraindications of SCS include but are not limited to spinal cord disorders, coagulopathy, local infection, sepsis, cognitive impairment causing an inability to handle and understand the usage of the device, and psychiatric conditions such as psychosis, schizophrenia, substance abuse, and severe depression or anxiety (16, 20). SCS electrode leads are typically placed within the epidural space at the T1–T2 level (6, 21). The procedure involves the placement of temporary percutaneous electrodes for an assessment period of 5–15 days to observe outcomes such as a 50% reduction in pain, paresthesia at the location of angina, and patient satisfaction (21). A permanent device is implanted if the desired outcome is achieved (6, 21).

Despite several theories, the exact mechanism of action of SCS underlying its therapeutic benefits in refractory anginal pain remains incompletely understood (19). The initial hypothesis regarding the mechanism of action of SCS was based on the gate control theory established by Melzack and Wall in 1965, proposing that one may prevent pain from being transmitted through the short A-delta and C fibers in the superficial dorsal horn by focusing stimulation on the large diameter A-beta fibers, causing paresthesia (22). Modern research conducted on animal subjects subsequently demonstrated that SCS leads to an increase in the release of gamma-amino acid (GABA), leading to the decrease of excitatory amino acids such as glutamate and aspartate in the dorsal horn (8, 21). Specifically, a study by Latif et al. observed that the effects of SCS are inhibited by antagonizing the GABA-b and adenosine A-1 receptors, suggesting that pain modulation by SCS arises in parts via these receptors (8). Thus, an increased inhibitory tone in the dorsal horn could explain the pain reduction experienced by patients with SCS.

In addition to the theories stated above, the mechanisms responsible for the anti-anginal and anti-ischemic properties of SCS still have to be thoroughly reported (17). Research indicates that the electrical current emitted by SCS in the epidural space may have an anti-anginal effect by reducing the hyperactivity of the sympathetic nervous system. This reduction in sympathetic tone leads to a decrease in oxygen demand in the myocardium, consequently resulting in anti-ischemic effects (8, 17, 21). In addition, a potent endogenous vasodilator known as calcitonin gene-related peptide (CGRP) was also demonstrated to be upregulated by SCS (8, 18, 19). The release of CGRP triggers the release of nitric oxide (NO), resulting in vascular smooth muscle relaxation, vasodilation, and a reduction of vasospasms, all contributing to the anti-ischemic effect of SCS (8, 18, 19). Croom et al. discovered that CGRP antagonists inhibited SCS-induced skin vasodilation in rats, clarifying the relationship between SCS and CGRP release (23). This finding implies that CGRP could be responsible for the clinical improvement of ischemic conditions following SCS (23). Thus, the reduction of sympathetic tone, consequently decreasing oxygen demand of the myocardium, in conjunction with CGRP and NO-induced vasodilation, appears to be the primary contributor to the anti-ischemic effect of SCS (8, 18, 21). The resulting effect is an increase in myocardial perfusion and a reduction of anginal pain (8, 18, 21).

A study by He et al. on the effects of SCS post–myocardial infarction in rabbit hearts revealed that SCS decreases the expression of inflammatory cytokines (TNF-α, IL-1β, IL-6, and NGF) and the infiltration of macrophages. This study suggests that SCS exhibits anti-inflammatory effects (24). Moreover, Wang et al. (25) reported the ability of SCS to decrease spinal neuroinflammation induced by cardiac myocardial infarction in rats. This effect is achieved by inhibiting spinal microglial p38 in the mitogen-activated protein kinase pathway (MAPK), which subsequently reduces the levels of proinflammatory mediators IL-1β and TNF-α in the spinal cord. Consequently, this leads to a reduction in angina-like pain (25). These studies suggest that cardiac function could be improved by SCS via multiple different mechanisms, suggesting that the use of SCS in cardiovascular diseases could benefit patients with conditions other than refractory angina pectoris, such as heart failure (24, 26).

### 3.2. Mechanism of action of SCS in heart failure

Most cases of HF are characterized by an imbalance of the autonomic nervous system, resulting in an increased sympathetic tone and a decreased parasympathetic tone (13). Although the effects of SCS on the autonomic nervous system were initially explored for RFA management, further research has found additional benefits, including the restoration of cardiac autonomic tone in patients with HF (13). Reducing sympathetic tone through SCS involves adjusting the activity of intrinsic afferent sensory cardiac neurons involved in sympathetic excitation. On the other hand, increasing parasympathetic tone through SCS involves slowing the sinus rate and lengthening the atrioventricular nodal conduction time and the ventricular refractory period (13, 27). The proposed effects of SCS on heart failure involve the modulation of sympathetic tone, drawing similarities with another form of neuromodulation utilized for heart failure, namely, vagus nerve stimulation (13, 28). More specifically, the stimulation of the auricular branch of the vagus nerves causes an increase in efferent vagal activity leading to a rise in parasympathetic tone and a fall in sympathetic tone, consequently leading to improvements in heart failure (28). Overall cardiac function and left ventricular function are thought to be improved by this effect of stimulation of the thoracic spinal cord, resulting in an increase in the LVEF (13). In a recent study by Ahmed et al., neuromodulation therapies for HF, including SCS, were found to reduce brain natriuretic peptide 32, suggesting that SCS decreases ventricular stress, a key component of heart failure (12). These novel discoveries raised new questions about the mechanism of action of spinal cord stimulation on the cardiovascular system (12). Finally, a prospective multi-center pilot trial by Tse et al. concluded that SCS is safe for patients with HF (13).

## 4. Discussion

Our patient experienced recurrent pericarditis of unknown etiology, causing refractory angina-like pain and heart failure with reduced left ventricular ejection fraction. SCS at the cervicothoracic junction led to significant clinical improvement in both aspects of his recurrent pericarditis. At the 6-month mark following SCS implantation, our patient discontinued hydromorphone and achieved freedom from narcotic use, with a reduction of 428 mg equivalent dose of morphine. In addition, our patient's LVEF improved by 5% without changes in medical therapy.

The beneficial effect of dorsal column stimulation on pain in our patient aligns with that of previous reports in the literature, consistent with Melzak et al.’s original description of the analgesic properties of SCS and Dermot et al.’s experiments, which first reported on the therapeutic benefits of SCS on pain reduction in refractory angina pectoris (7, 22). However, neither the gate control theory explained by Melzak et al. nor its impact on inhibitory neurotransmitters as shown by Latif et al. could solely account for our patient's clinical improvement of heart failure (8).

Moreover, neither theory could explain the efficacy of SCS in the treatment of pericarditis, which is an inflammatory condition (8, 22). It has been shown that inflammation through the NLRP3 inflammasome pathway is the key to pericarditis pathophysiology (29). Thus, this report supports the notion that SCS modulates inflammatory cytokine secretion, leading to an anti-inflammatory effect (24, 25). This effect is known to provide analgesia in refractory angina-like pain but could also relieve perimyocardial inflammation, longitudinally leading to improved left ventricular function in our patient.

The improvement of our patient's LVEF following SCS is consistent with reports by Tse et al. in cases of heart failure, where 88% of study participants reported a minimal increase in baseline LVEF of 5% (13). In our specific case of refractory pericarditis, two distinct yet non-exclusive mechanisms could underlie LVEF improvement, namely, the modulation of sympathetic tone as described in heart failure (12, 13) and the resolution of perimyocardial inflammation through SCS-mediated reduction of proinflammatory cytokine secretion (24, 25).

Interestingly, there are some reported cases of stellate ganglion block in acute and recurrent pericarditis (30, 31). While SGB was performed in our patient in accordance with its indications in refractory angina-like pain (32), research demonstrates potent anti-inflammatory properties of SGB, leading to a reduction of proinflammatory cytokines IL-1β, IL-6, and TNF-α (33). Moreover, SGB inhibits the activity of both central and peripheral sympathetic nerves, correcting the pathological hyper-function of sympathetic activity and restoring autonomic tone (33). Therefore, the temporary effectiveness of stellate ganglion block in our patient may have resulted from similar mechanisms of action as those discussed above in relation to spinal cord stimulation.

Our hypothesis with regard to the therapeutic benefits of SCS in refractory pericarditis involves the synergy of numerous simultaneous effects originating from dorsal column stimulation. These effects encompass increased inhibitory tone in the dorsal horn, modulation of proinflammatory cytokine secretion, reduced sympathetic tone, and vasodilation by CGRP and NO release. In consequence, our patient potentially benefited from the analgesic, anti-ischemic, and anti-inflammatory properties of SCS, leading to a reduction in angina-like pain. Likewise, the anti-inflammatory effects leading to a resolution of perimyocardial inflammation and/or modulation of sympathetic tone by SCS could underlie our patient's increased LVEF.

A notable limitation of this study arises from our patient's substantial 80% improvement in low back pain compared with the 60% reduction in chest pain. This difference could have influenced the patient's perception of overall pain relief and likely contributed to the successful reduction in narcotic use following SCS implantation. Moreover, as with every case report, the primary limitation is the lack of generalizability and the inability to demonstrate a cause-and-effect relationship.

## 5. Conclusion

We report the first case of SCS for pericarditis in the absence of coronaropathy in a patient with pain mimicking the presentation of refractory angina pectoris and heart failure. Despite the absence of vascular disorder, we provided satisfactory pain control and improved left ventricular ejection fraction. A larger randomized trial to confirm our findings regarding SCS use in cardiovascular pathologies other than RFA and HF could expand the utility of SCS in modern medicine.

## Data Availability

The original contributions presented in the study are included in the article, further inquiries can be directed to the corresponding author.
